# Exploring the alignment between clinician-reported assessment of social autonomy and patient-reported assessment of quality of life in mood disorders: a cross-sectional study

**DOI:** 10.3389/fpsyt.2024.1466856

**Published:** 2025-07-04

**Authors:** Alexandre Fraichot, Sophie Favre, Françoise Jermann, Hélène Richard-Lepouriel

**Affiliations:** Department of Psychiatry, University Hospitals of Geneva, Geneva, Switzerland

**Keywords:** social autonomy, quality of life, patient assessment, clinician assessment, mood disorder, agreement

## Abstract

**Introduction:**

Patient-reported quality of life reflects subjective factors such as well-being and autonomy, while clinicians may focus on functional capabilities. Understanding the factors behind the alignment or discordance between these assessments can help comprehend patients’ values and social contexts.

**Methods:**

This study explored the agreement between clinician-reported assessment of social autonomy and patient-reported assessment of quality of life in 92 adult participants with a mood disorder. Validated scales were used to measure the severity of depression, hypomania, quality of life, social autonomy, and internalized stigma.

**Results:**

Sociodemographic and clinical variables were compared between different groups using ANOVAs and chi-square tests. The results indicated that individuals with good social autonomy and quality of life had lower self-stigma scores. Those with low social autonomy and quality of life were less likely to be employed. The group with discordant scores between social autonomy and quality of life did not significantly differ from the other concordant groups in terms of sociodemographic and clinical variables.

**Discussion:**

The study suggests that mental health professionals should consider the association between clinician-reported and patient-reported assessments and their correlates before tailoring specific interventions.

## 1 Introduction

Mood disorders are categorized as serious mental illnesses that can significantly impact a person’s life. These disorders include depression and bipolar disorder. It is estimated that 21.4% of adults in the United States experience a mood disorder at some point in their life. Out of these, 16.9% experience major depressive disorder (MDD), 4.4% experience bipolar disorder (BD), and 2.5% experience dysthymia. These disorders can cause varying degrees of impairment, with an estimated 45% experiencing severe impairment, 40% experiencing moderate impairment, and 15% experiencing mild impairment (1). Additionally, mood disorders can be associated with other mental health conditions, such as anxiety disorders, personality disorders, substance use disorders, attention-deficit/hyperactivity disorders, and eating disorders (2).

The concept of Quality of Life (QOL) measures a person’s well-being across four dimensions: physical health, mental health, social health, and functional health (3). The World Health Organization defines QOL as an individual’s perception of their position in life within the context of their culture and value systems, encompassing their goals, expectations, standards, and concerns (4). This multidimensional concept has been assessed using various scales and studied in specific patient groups. A systematic review and meta-analysis on QOL in euthymic patients with BD showed that QOL was lower in BD patients than in healthy controls, and more extended periods of euthymia were associated with better QOL scores. In another study that compared QOL in euthymic patients with MDD and BD, results showed that QOL scores were lower in both MDD and BD patients than in the general population and lower for MDD patients than those with BD (5). Additionally, QOL was lower in individuals with anxiety and depression compared to healthy controls both before onset and after remission, and QOL further decreased during the course of these disorders (6).

There is a limited understanding of the correlation between subjective QOL and objective measures of psychosocial functioning. A study on postpartum depression found a significant association between patient-reported outcomes and clinician-reported outcomes. Clinical improvements were associated with patient-reported symptom relief (7). Additionally, a longitudinal study examined how the relationship between subjective life satisfaction and objective functional outcomes in individuals with a mood disorder evolved over 7-8 years (8). The study found that for patients with nonpsychotic depression, their subjective life satisfaction was closely related to their global functioning, work performance, and social adjustment at each follow-up. However, this correlation was not observed for patients with BD or psychotic depression.

The research highlighted the complex connection between an individual’s perception of well-being and their actual functioning in mood disorders. Personal life satisfaction may not always align with measurable functional improvements, possibly due to reduced insight, demoralization, or changes in life expectancy over time. Subjective life satisfaction and objective functional outcomes are separate but interconnected aspects of well-being for individuals with mood disorders. Therefore, it’s essential to consider both subjective and objective factors when assessing the overall well-being of individuals with mood disorders. Effective treatment should improve functional outcomes and life satisfaction for better patient results.

Furthermore, there have been doubts about how accurately self-report measures reflect life satisfaction in severe mood disorders. Establishing standards in patient-centered outcomes research aims to promote the proper use of patient-reported outcomes and improve the efficiency and effectiveness of healthcare delivery (9). By considering both patient-reported and clinician-assessed outcomes, treatment plans can be tailored based on the clinical assessment and the patient’s individual experiences and preferences, thus promoting shared decision-making.

Autonomy refers to a person’s ability to lead a meaningful life (10). The authors suggested that reduced autonomy is a disregarded aspect of mental illness and may be negatively affected by stigma. A cross-sectional study involving 104 patients with BD type I, using the self-reported social autonomy scale (EAS) and clinicians’ assessments of the Global Assessment of Functioning (GAF) scores, found that most patients (82.3%) had social autonomy scale scores below 59, indicating impairment in social autonomy (11). Additionally, almost half of the participants (41.4%) had overall impairment with GAF scores below 70. The study also showed a negative correlation between global functioning and social autonomy across five dimensions. Factors contributing to social impairment included recent depressive episodes, multiple hospitalizations, and treatment-related side effects (11).

Several studies have indicated a strong correlation between autonomy and QOL. For instance, individuals with intellectual disabilities with a higher degree of autonomy reported better QOL (12). This relationship has also been explored in the context of rehabilitation goals, emphasizing combining independence, autonomy, and social engagement to enhance QOL (13).

The research suggests a connection between decreased autonomy and a decline in QOL for individuals (11–13). However, in our clinical experience, we have observed cases where a significant reduction in autonomy was associated with a high QOL. Observing cases where reduced autonomy is linked to high QOL is important as it challenges assumptions about the relationship between autonomy and well-being. It broadens the understanding of QOL factors, guides personalized care, and supports more patient-centered healthcare approaches. To bridge the gap in understanding the characteristics of persons with concordant and discordant variations of autonomy and QOL, this study compared clinicians’ assessments of social autonomy with the subjective QOL of individuals living with a mood disorder. Our main goal was to assess sociodemographic and clinical factors in persons with mood disorders to understand the characteristics of those who experience parallel or non-aligned variations between autonomy and QOL. The primary assumption was that when exploring the agreement between clinician-reported assessment of social autonomy and patient-reported assessment of quality of life in mood disorders, there would be three different groups: one where the assessments would parallel (either toward the negative or the positive) and one where the assessments would be discordant. This assumption is rooted in the logical construct that when two perspectives are present, there can be either concordance or discordance between them. In this study, concordance can have two different values.

Additionally, self-stigma is when individuals internalize negative stereotypes about mental health, which can lead to feelings of shame, guilt, and reduced self-worth. This often leads to lower self-esteem and a reluctance to seek help (14). Feeling unworthy can result in reduced social engagement, which may affect self-reported quality-of-life assessments, potentially underestimating one’s well-being. Clinicians who assess autonomy based on interactions and engagement might report higher levels of social autonomy than patients feel they possess, creating a disconnect. Patients may also develop coping strategies to deal with discrimination and prejudice, described as stigma resistance (15), leading to differences between their lived experience and how clinicians perceive their autonomy. The secondary assumption was that self-stigma would be associated with the concordant groups, with high self-stigma associated with the negative concordant group and low self-stigma with the positive concordant group. This assumption is based on previous studies (14, 15) demonstrating self-stigma’s impact on subjective evaluations. Individuals with high self-stigma report a low quality of life, which reinforces negative self-perceptions, feelings of isolation, and global functioning that the clinicians can assess. Conversely, individuals who report a high quality of life and low self-stigma are less affected by societal stigma, leading to more positive self-assessment and global functioning.

## 2 Materials and methods

### 2.1 Study setting and sampling

Ninety-two participants were recruited in the outpatient mood disorder unit of Geneva’s University Hospitals, Switzerland.

### 2.2 Inclusion criteria

Inclusion criteria were (1): a diagnosis of mood disorders (Major Depressive Disorder, Bipolar Disorder), (2) an age of 18 years or above, and (3) fluency in French.

Diagnostic was made by a best estimate procedure including a thorough anamnesis (medical histories, family history, onset of the disorder, and previous treatments) by a psychiatrist. The procedure also encompassed a confrontation with the results of the 7^th^ version of a semi-structured questionnaire, the International Neuropsychiatric Interview (MINI) (16), that was developed to assess the Diagnostic and Statistical Manual of Mental Disorders 5TH edition (17) criteria and was completed by a trained psychologist. The diagnosis assessment was completed by a psychiatric nurse evaluating the patients’ functioning. Self-report questionnaires finalized the process. Patients with a mood disorder were included in the study.

### 2.3 Instruments

#### 2.3.1 Clinician assessment scales

##### 2.3.1.1 The mini international neuropsychiatric interview

The MINI (16) is a brief, structured diagnostic interview designed for the most common DSM-5 and ICD-11 psychiatric disorders. It is used in clinical settings and has demonstrated good interrater and test-retest reliability and validity. The MINI is widely used and can be particularly relevant in multicenter clinical trials because it is a standardized, easy-to-administer, and validated tool available in several languages.

##### 2.3.1.2 The Montgomery-Asberg depression rating scale

The MADRS (18, 19) is commonly used to evaluate the severity of depressive symptoms. It is a 10-item scale completed by a clinician. Each item is scored from 0 to 6. A score ranging from 0 to 6 indicates no depression, 7 to 19 indicates “mild depression,” 20 to 34 indicates “moderate depression,” and 35 and greater indicates “severe depression.” (20).

##### 2.3.1.3 The young mania rating scale

The YMRS (21, 22) is commonly used to evaluate the severity of depressive symptoms. It is a 10-item scale completed by a clinician. Each item is scored from 0 to 6. A score ranging from 0 to 6 indicates no depression, 7 to 19 indicates “mild depression,” 20 to 34 indicates “moderate depression,” and 35 and greater indicates “severe depression) is a clinical interview scale used to assess the severity of mania. It consists of 11 items. Four items (irritability, speech, thought content, and disruptive/aggressive behavior) are graded on a scale of 0 to 8, while the remaining seven are graded on a scale of 0 to 4. A score ranging from 0 to 5 indicates euthymia, 6 to 14 indicates hypomania and a score of 15 or more indicates mania.

##### 2.3.1.4 L’Echelle d’Autonomie Sociale

The EAS (23) is a French-language clinician evaluation scale that assesses the level of social autonomy of people with severe psychiatric disorders on five dimensions of daily life. It is a 17-item scale. The items are rated from 0 to 6, with the lowest score indicating the best performance (total score range 0-102). The five subscales are: self-care (items 1 to 3), management of daily life (items 4 to 7), management of resources (items 8 to 10), relationships with the outside world (items 11 to 14), and emotional life and social relationships (items 15 to 17).

#### 2.3.2 Self-assessment scales

##### 2.3.2.1 The internalized stigma of mental illness scale

The ISMI Scale (24, 25) is a commonly used self-assessment questionnaire that measures self-stigma. It consists of 29 items rated on a four-point Likert scale. It is divided into five subscales: Alienation (6 items), Stereotype Endorsement (7 items), Perceived Discrimination (5 items), Social Withdrawal (6 items), and Stigma Resistance (5 items).

The Alienation subscale measures the patient’s feeling of social exclusion or not being entirely accepted in society due to their mental illness. The Stereotype Endorsement subscale measures the degree to which the individual agrees with social stereotypes about people with mental illness. The Perceived Discrimination subscale measures the patient’s perception of being discriminated against because of their mental illness. The Social Withdrawal scale measures the patient’s perception of avoiding social interactions due to fear of rejection caused by their mental illness. The Stigma Resistance subscale measures the patient’s ability to withstand self-stigma. The items in this subscale are reverse-coded, meaning that a high score indicates a lower level of self-stigma.

##### 2.3.2.2 The Brief-World Health Organization quality of life

The Brief-WHOQOL (4) is a widely used self-assessment questionnaire that evaluates an individual’s subjective well-being and functioning in various aspects of life. It comprises 26 items covering 12 dimensions: physical health, sleep, mood, cognition, leisure, social relationships, spirituality, finances, household, self-esteem, independence, and identity. Each dimension is rated on a five-point Likert scale from 1 to 5, where higher scores indicate better QOL. The total score ranges from 12 to 60 and is divided into 4 subscales: “physical health,” “psychological well-being,” “social relations,” and “environment.”

### 2.4 Ethical considerations

The Swiss Association of Research Ethics Committees (Swissethics) approved the study (Approval No: CCER_2023-02360). The study included adults diagnosed with mood disorders. All participants provided informed consent. There was no compensation for participation.

### 2.5 Data collection

In 2021, every patient undergoing a diagnostic assessment for a mood disorder was asked to sign an informed consent form. Only those who agreed to include their data in an anonymized research protocol were selected for the sample.

### 2.6 Data analysis

#### 2.6.1 Definition of concordant (positive and negative) and discordant groups

In exploratory analyses, median splits are used to identify distinct patterns. Using median splits has advantages because they provide a straightforward way to categorize continuous variables, making it easier to visualize findings and enhance interpretability. However, using median splits can lead to a potential loss of information and reduced statistical power. Iacobucci et al. (26, 27) have advocated that median splits can be a reliable approach after carefully considering the research design and the study’s goal. Moreover, recent studies have used this approach (28–30). Cross-sectional studies can effectively utilize median splits, particularly for identifying and comparing groups, which was the goal of this study. To test our assumptions, median splits were used to categorize participants into high and low groups on both the patient-reported and clinician-reported assessment scales. Three groups were created using a median split of social autonomy scores, measured with the EAS and QOL scores calculated with the Brief-WHOQOL, with median scores of 16.5 and 53.3, respectively. Persons reporting good social autonomy and QOL constituted the positive concordant group, and persons reporting low social autonomy and poor QOL constituted the negative concordant group. Persons reporting either low social autonomy but good QOL or good social autonomy, but poor QOL constituted the discordant group.

#### 2.6.2 Statistical analysis

Statistics were computed using SPSS version 25 (IBM, Armonk, NY). Questionnaire scores were normally distributed (all skewness values were between − 0.43 and 0.86, and kurtosis values were between – 1.41 and 0.07) except the YMRS score (skewness 3.40; kurtosis 12.50). Differences between groups for demographic and clinical variables were tested with ANOVAs and chi-square tests as appropriate. One-way ANCOVAs with MADRS as a covariate were performed with ISMI subscales as dependent variables and groups as independent variables. The significance level was set at *p* < 0.05.

## 3 Results

### 3.1 Characteristics of the sample

The sample comprised 92 participants divided into three groups (the positive concordant group, n=34, the negative concordant group, n=33, and the discordant group, n=25). Demographic and clinical data are presented in **Table 1**. Regarding demographic data, there are no significant differences between groups except for professional status, and marital status, with a higher percentage of people working or studying in the positive concordant group than in the negative concordant group (chi-square p=.08) and persons more with partners in the concordant group. Regarding the clinical data, groups were only different concerning the MADRS score, with people in the positive concordant group showing a lower score meaning fewer depressive symptoms than people in the discordant group, which showed lower scores, fewer depressive symptoms, than people in the negative concordant group (all *post hoc* Tukey HSD tests <.05).

**Table 1:** Socio-demographic and clinical data for the concordant (positive and negative) and discordant groups.

	Total sample (n=92)	Positive concordant group (n=34)	Negative concordant group (n=33)	Discordant group (n=25)	Chi-Square or ANOVA as appropriate (p-value)
Socio-demographics					
Age (mean-SD)	40.2 (12.3)	40.3 (11.3)	40.9 (12.1)	39.4 (14.1)	.90
Gender (% female)	54%	50%	52%	64%	.52
Marital status					.14
- Single	41%	21%	58%	48%	
- Married/partnered	37%	50%	27%	32%	
- Separated/divorced/widowed	22%	29%	15%	20%	
Education					.32
- Mandatory school or less	11%	3%	19%	12%	
- Apprenticeship or highschool	29%	33%	22%	32%	
- University or similar	60%	64%	59%	56%	
Professional status					
- Working or studying	61%	79% ^a^	42% ^b^	60%	.008
Clinical data					
Mood disorder diagnosis					.09
- Major depressive disorders (F32, F33 and F34)	64%	50%	70%	76%	
- Bipolar disorers (F31)	36%	50%	30%	24%	
Disorder duration: nb of years since first mood episode (mean, SD)	15.3 (12.6)	14.7 (13.2)	15.0 (11.2)	16.5 (13.8)	.86
Lifetime presence of suicide attempts (%)	15%	9%	24%	12%	.19
MADRS	14.37 (11.5)	8.41 (7.2) ^a^	19.67 (12.5) ^b^	15.64 (9.4) ^c^	<.001
YMRS	0.60 (1.5)	0.68 (1.7)	0.41 (1.0)	0.71 (1.8)	.74

MADRS data are missing for 3 people in the discordant group; YMRS data are messing for 3 people in the positive concordant group, for 6 people in the negative concordant group and for one person in the discordant group. Groups with different superscript letters (e.g., a, b) in the same row are significantly different from each other based on *post hoc* tests (*post hoc* Tukey HSD tests).

### 3.2 Statistical analysis

The means (SD) for self-stigma (ISMI) subscales are presented in **Table 2**. Differences between groups were explored using ANCOVAs for ISMI subscales with MADRS score as a covariate. Regarding the ISMI subscales, results showed a significant group effect for alienation (p<.01), stereotype endorsement (p<.001), discrimination (p<.01), social withdrawal (p<.01) but not for stigma resistance (p=.32). For alienation, stereotype endorsement and discrimination, *post hoc* tests (Bonferroni) showed significantly lower scores, in the positive concordant group than in the negative concordant and discordant groups (all p<.05) meaning a lower tendency to have a subjective experience of being less than a full member of society or having a ‘spoiled identity’ (alienation score), a lower agreement with common stereotypes about people with mental illness (stereotype endorsement), feeling less discriminated by others (discrimination). For social withdrawal, *post hoc* tests showed a significant difference between the positive concordant group and negative concordant group with the positive concordant group showing lower scores than negative concordant group (p<.05) (see **Table 2**), meaning a lower tendency to withdraw from social interaction.

**Table 2:** Self-stigma (ISMI) data (mean-SD) for the concordant (positive and negative) and discordant groups.

Variables	Total sample	Positive concordant group (Pos)	Negative concordant group (Neg)	Discordant group (Dis)	ANCOVA		Paiwise comparisons	Mean differnce	SE	Adjusted p
	(n=92)	(n=34)	(n=33)	(n=25)	F	p				
ISMI										
Alienation	2.51 (0.8)	2.00 (0.8)	2.88 (0.5)	2.73 (0.7)	10.45	<.001	Pos vs Neg	-0.79	.19	<.001
							Pos vs Dis	-0.68	.19	<.01
							Neg vs Dis	0.11	.19	1.0
Stereotype endorsement	1.75 (0.5)	1.36 (0.4)	1.97 (0.5)	1.93 (0.5)	10.66	<.001	Pos vs Neg	-0.57	.14	<.001
							Pos vs Dis	-0.54	.14	<.01
							Neg vs Dis	0.02	.13	1.0
Discrimination	1.89 (0.7)	1.49 (0.5)	2.17 (0.7)	2.01 (0.6)	6.41	.003	Pos vs Neg	-0.60	.17	<.01
							Pos vs Dis	-0.46	.18	<.05
							Neg vs Dis	0.14	.17	1.0
Social withdrawal	2.19 (0.8)	1.76 (0.6)	2.57 (0.7)	2.27 (0.7)	5.80	.004	Pos vs Neg	-0.62	.18	<.01
							Pos vs Dis	-0.39	.19	.13
							Neg vs Dis	0.23	.19	.65
Resistance	2.43 (0.5)	2.22 (0.5)	2.61 (0.5)	2.41 (0.5)	1.16	.319	Pos vs Neg	-0.21	.14	.40
							Pos vs Dis	-0.11	.14	1.0
							Neg vs Dis	0.10	.14	1.0

For the total sample, data for the alienation score is missing for 3 people; data for the stereotype endorsement is missing for 6 people; data for the discrimination score is missing for 5 people; data for the social withdrawal score is missing for 1 person; data for the resistance score is missing for 4 people.

## 4 Discussion

This study was based on the hypothesis that differences between clinician and patient assessments can provide valuable information for improving the quality of care and may indicate a need for personalized interventions. Our goal was to examine whether different groups share similarities and differences compared to other groups. We specifically focused on the sociodemographic and clinical characteristics of patients with mood disorders and their association with the different groups.

In the positive concordant group, self-stigma scores were lower on different subscales than the other groups. The scores were lower on alienation, stereotype endorsement, perceived discrimination, and social withdrawal. However, another study showed that both alienation and stereotype endorsement had direct effects on QOL in persons with schizophrenia (31). These differences illustrate a consistent interaction between self-stigma and QOL. Individuals in the positive concordant group may find that their decisions align with their values and lead to improved well-being, including greater satisfaction in relationships and a reduced sense of marginalization. This suggests that individuals in the positive concordant group may feel more included in society.

Employment rates were lower in the negative concordant group, and scores on most self-stigma measures were higher. A review on unemployment and stigma (32) showed that people with mental illness were more likely to be unemployed and that they often faced hostility and reduced responsibilities, which may result in increasing self-stigma and disability. This suggests that this negative concordant group may encounter more social and emotional challenges, impacting their QOL and autonomy. The negative concordant group comprised individuals with low social autonomy, possibly making them less empowered to make decisions and more restricted. They also had lower QOL scores, which may indicate experiences of challenges in various aspects, such as social isolation or lack of opportunities and internalization of negative societal attitudes, resulting in feelings of shame and doubt. A systematic review and meta-analysis also described a statistically significant association between higher stigma and lower subjective QOL in persons with psychosis (33). Their narrative summary of 12 studies suggested that psychological mechanisms relating to self-concept and social networks may play a crucial mediating role in the association between stigma and QoL in psychosis. These findings underscore the pervasive nature of stigma on QOL in persons with a mental illness and suggest that interventions should target reducing the negative impact of stigma on QOL. Liu et al. (2024) (34) conducted a cross-sectional study that showed a connection between self-stigma and QOL in individuals with schizophrenia. They emphasized that various factors, such as coping strategies like avoidance and low self-esteem, are linked to higher levels of self-stigma. The results of the study emphasized the importance of addressing self-stigma in individuals with schizophrenia to improve their overall QOL. This also applies to individuals with BD (35). Their review described how stigma significantly impacts those living with BD. Many individuals experience negative consequences stemming from stereotypes, prejudice, and discrimination associated with their condition. Public stigma was found to be associated with greater functional impairment, heightened anxiety, and poorer work-related outcomes. Meanwhile, self-stigma correlated with lower levels of functioning across various domains, as well as increased depressive and anxiety symptoms. Additionally, Kumari et al. (2023) (36) demonstrated that nurse-led brief psychoeducation can reduce self-stigma among individuals with schizophrenia and affective disorders. The nurse-led interventions aimed to empower clients, reduce self-stigma, and enhance quality of life. They involved brief psychoeducation addressing self-stigma among individuals with schizophrenia and affective disorders. The key components of these interventions included providing information about mental health conditions, the nature of stigma, and its effects on self-esteem and recovery, coping strategies, ways to challenge negative self-perceptions and societal attitudes, fostering a supportive atmosphere where clients can share experiences and feelings, promoting open dialogue, and follow-up assessments to evaluate the effectiveness of the intervention and support ongoing recovery. While further evidence is needed, this study also highlights the potential of brief nurse-led interventions to enhance treatment.

We had expected the main differences to be between the concordant and discordant groups. Surprisingly, there were no significant differences in sociodemographic factors between the groups. However, when clinical variables were considered to compare the discordant and non-discordant groups, the results showed that the disparities were primarily between the concordant groups rather than the discordant group. These differences were in various areas, including employment and ISMI subscales (except for stigma resistance). The study also found that individuals with higher levels of stigma resistance, which is the ability to counteract stigma, tend to have improved social functioning (37). Stigma resistance may be a crucial factor for recovery (37). However, our results show that this subscale was not associated with just one group.

The presence of a discordant group (27% of the sample) suggests that there may be a complex relationship between social autonomy and QOL. Our findings show that despite limitations in their autonomy, some individuals may still experience overall satisfaction. Other factors, such as having a strong support system, effective coping strategies, and a resilient mindset, may also play a role in this complex interaction. On the other hand, some individuals may have high social autonomy while experiencing low QOL. Factors such as unmet expectations or institutionalized inequality may contribute to this dynamic. Further research is necessary to support these hypotheses.

The difference between how patients perceive their situation and how clinicians evaluate it using an assessment scale may stem from variations in identifying impairments or underlying factors that the clinician or assessment scale may not fully recognize. Not only self-stigma but also personal factors such as personality traits, coping mechanisms, social support networks, cultural background, and life circumstances can influence these perceptions. When there is a gap between the patient’s view of their QOL and the clinician’s evaluation of autonomy, it is important to understand the reasons behind the patient’s perception. Acknowledging and addressing these differences can lead to collaborative and personalized treatment planning.

### 4.1 Strengths and limitations

Patients with low autonomy and low QOL scores also display higher self-stigma scores. However, as this is a cross-sectional study, no causal relationship can be established. Longitudinal studies are needed to establish cause-and-effect relationships and identify changes over time. Moreover, the non-probability sampling methods are a risk for a sampling bias, limiting the inferences. In this study, the positive concordant group had more participants who worked or studied than the negative concordant group. These differences could influence some outcomes, such as the association with self-stigma, QOL, and social autonomy. Future research should investigate these specific associations further. One limitation of the current study is that it lacked stratification based on diagnosis, which may lead to biased results. The characteristics of one group may distort the findings related to others, and the findings may also not be generalizable. However, since this is exploratory research, the goal was to identify general trends. In this case, stratification is less critical. Moreover, the outcomes were not expected to vary significantly by diagnosis. This study did not include specific clinical outcomes, such as the frequency of hospitalizations, the presence of disabling comorbidities, the seasonal pattern of mood episodes, or the presence of psychotic symptoms during mood episodes. This is because this study’s main aim was to explore the association between clinicians’ and patients’ agreement on assessments of social autonomy and quality of life. However, future studies could consider incorporating these clinical features to investigate their impact on this association further.

Finally, although the small size of the discordant group (n=13 with low social autonomy and good QOL; n=12 with good social autonomy and poor QOL) did not allow for subgroup specification, it did not affect the overall findings.

## 5 Conclusion

The results of this study emphasize the importance of determining if the subjective and objective measures of functioning align and evaluate self-stigma in individuals with mood disorders. Considering the agreement between clinicians and patients, along with self-stigma, can help identify which elements of psychoeducation are most crucial. Mental health professionals should particularly pay attention to patients with low social autonomy, who perceive their QOL as poor and experience high self-stigma. These patients may benefit from tailored psychoeducation programs to address their specific needs.

## Data Availability

The datasets presented in this study can be found in online repositories. The names of the repository/repositories and accession number(s) can be found below: https://yareta.unige.ch/home.
